# Updating *QR* factorization procedure for solution of linear least squares problem with equality constraints

**DOI:** 10.1186/s13660-017-1547-0

**Published:** 2017-11-13

**Authors:** Salman Zeb, Muhammad Yousaf

**Affiliations:** Department of Mathematics, University of Malakand, Dir (Lower), Chakdara, Khyber Pakhtunkhwa Pakistan

**Keywords:** 65-XX, 65Fxx, 65F20, 65F25, *QR* factorization, orthogonal transformation, updating, least squares problems, equality constraints

## Abstract

In this article, we present a *QR* updating procedure as a solution approach for linear least squares problem with equality constraints. We reduce the constrained problem to unconstrained linear least squares and partition it into a small subproblem. The *QR* factorization of the subproblem is calculated and then we apply updating techniques to its upper triangular factor *R* to obtain its solution. We carry out the error analysis of the proposed algorithm to show that it is backward stable. We also illustrate the implementation and accuracy of the proposed algorithm by providing some numerical experiments with particular emphasis on dense problems.

## Introduction

We consider the linear least squares problem with equality constraints (LSE)

1 $$ \min_{x} \Vert Ax-b \Vert _{2},\quad \text{subject to } Bx=d, $$

where $A \in R^{m\times n}$, $b \in R^{m}, B\in R^{p \times n}$, $d\in R^{p}$, $x\in R^{n}$ with $m+p\geq n\geq p$ and $\Vert \cdot \Vert _{2}$ denotes the Euclidean norm. It arises in important applications of science and engineering such as in beam-forming in signal processing, curve fitting, solutions of inequality constrained least squares problems, penalty function methods in nonlinear optimization, electromagnetic data processing and in the analysis of large scale structure [1–8]. The assumptions

2 $$ \operatorname{rank}(B)=p \quad\text{and} \quad\operatorname{null}(A)\cap\operatorname{null}(B)=\{ 0\} $$

ensure the existence and uniqueness of solution for problem (1).

The solution of LSE problem (1) can be obtained using direct elimination, the nullspace method and method of weighting. In direct elimination and nullspace methods, the LSE problem is first transformed into unconstrained linear least squares (LLS) problem and then it is solved via normal equations or *QR* factorization methods. In the method of weighting, a large suitable weighted factor *γ* is selected such that the weighted residual $\gamma(d-Bx)$ remains of the same size as that of the residual $b-Ax$ and the constraints is satisfied effectively. Then the solution of the LSE problem is approximated by solving the weighted LLS problem. In [9], the author studied the method of weighting for LSE problem and provided a natural criterion of selecting the weighted factor *γ* such that $\gamma\geq \Vert A \Vert _{2}/ \Vert B \Vert _{2} \epsilon _{M}$, where $\epsilon_{M}$ is the rounding unit. For further details as regards methods of solution for LSE problem (1), we refer to [2, 3, 7, 10–13].

Updating is a process which allow us to approximate the solution of the original problem without solving it afresh. It is useful in applications such as in solving a sequence of modified related problems by adding or removing data from the original problem. Stable and efficient methods of updating are required in various fields of science and engineering such as in optimization and signal processing [14], statistics [15], network and structural analysis [16, 17] and discretization of differential equations [18]. Various updating techniques based on matrix factorization for different kinds of problems exist in the literature [3, 11, 13, 19–22]. Hammarling and Lucas [23] discussed updating the *QR* factorization with applications to LLS problem and presented updating algorithms which exploited LEVEL 3 BLAS. Yousaf [24] studied repeated updating based on *QR* factorization as a solution tool for LLS problem. Parallel implementation on GPUs of the updating *QR* factorization algorithms presented in [23] is performed by Andrew and Dingle [25]. Zhdanov and Gogoleva [26] studied augmented regularized normal equations for solving LSE problems. Zeb and Yousaf [27] presented an updating algorithm by repeatedly updating both factors of the *QR* factorization for the solutions of LSE problems.

In this article, we consider the LSE problem (1) in the following form:

3 $$ \min_{x{(\gamma)}} \left \Vert \begin{pmatrix} \gamma B \\ A \end{pmatrix}x-\begin{pmatrix} \gamma d \\ b \end{pmatrix} \right \Vert _{2}, $$

which is an unconstrained weighted LLS problem where $\gamma\geq \Vert A \Vert _{2}/ \Vert B \Vert _{2} \epsilon _{M}$ given in [9] and approximated its solution by updating Householder *QR* factorization. It is well known that Householder *QR* factorization is backward stable [11, 12, 28, 29]. The conditions given in (2) ensure that problem (3) is a full rank LLS problem. Hence, there exist a unique solution $x(\gamma)$ of problem (3) which approximated the solution $x_{\mathrm{LSE}}$ of the LSE problem (1) such that $\lim_{\gamma \rightarrow\infty} x(\gamma)=x_{\mathrm{LSE}}$. In our proposed technique, we reduced problem (3) to a small subproblem using a suitable partitioning strategy by removing blocks of rows and columns. The *QR* factorization of the subproblem is calculated and its *R* factor is then updated by appending the removed block of columns and rows, respectively, to approximate the solution of problem (1). The presented approach is suitable for solution of dense problems and also applicable for those applications where we are adding new data to the original problem and *QR* factorization of the problem matrix is already available.

We organized this article as follows. Section 2 contains preliminaries related to our main results. In Section 3, we present the *QR* updating procedure and algorithm for solution of LSE problem (1). The error analysis of the proposed algorithm is provided in Section 4. Numerical experiments and comparison of solutions is given in Section 5, while our conclusion is given in Section 6.

## Preliminaries

This section contains important concepts which will be used in the forthcoming sections.

### The method of weighting

This method is based on the observations that while solving LSE problem (1) we are interested that some equations are to be exactly satisfied. This can be achieved by multiplying large weighted factor *γ* to those equations. Then we can solve the resulting weighted LLS problem (3). The method of weighting is useful as it allows for the use of subroutines for LLS problems to approximate the solution of LSE problem. However, the use of the large weighted factor *γ* can compromise the conditioning of the constrained matrix. In particular, the method of normal equations when applied to problem (3) fails for large values of *γ* in general. For details, see [3, 11–13].

### *QR* factorization and householder reflection

Let

4 $$ X=\mathit{QR} $$

be the *QR* factorization of a matrix $X \in \mathcal {R}^{m\times n}$ where $Q\in \mathcal {R}^{m\times m}$ and $R\in \mathcal {R}^{m\times n}$. This factorization can be obtained using Householder reflections, Givens rotations and the classical/modified Gram-Schmidt orthogonalization. The Householder and Givens *QR* factorization methods are backward stable. For details as regards *QR* factorization, we refer to [11, 12].

Here, we briefly discuss the Householder reflection method due to our requirements. For a non-zero Householder vector $v\in\mathcal {R}^{n}$, we define the matrix $H\in \mathcal {R}^{n\times n}$ as

5 $$ H=I_{n}- \tau v v^{T},\quad \tau=\frac{2}{v^{T} v}, $$

which is called the Householder reflection or Householder matrix. Householder matrices are symmetric and orthogonal. For a non-zero vector *u*, the Householder vector *v* is simply defined as

$$v= u \pm \Vert u \Vert _{2} e_{k}, $$

such that

6 $$ Hu=u- \tau v v^{T}u= \mp\alpha e_{k}, $$

where $\alpha= \Vert u \Vert _{2}$ and $e_{k}$ denotes the *k*th unit vector in $\mathcal{R}^{n}$ in the following form:

$$e_{k}(i)= \textstyle\begin{cases} 1 & \mbox{if } i=k,\\ 0 & \mbox{otherwise}. \end{cases} $$

In our present work given in next section, we will use the following algorithm for computation of Householder vector *v*. This computation is based on Parlett’s formula [30]:

$$ v=u_{1}- \Vert u \Vert _{2}=\frac{u_{1}^{2}- \Vert u \Vert _{2}^{2}}{x_{1}+ \Vert u \Vert _{2}}= \frac {- \Vert u \Vert _{2}^{2}}{u_{1}+ \Vert u \Vert _{2}}, $$

where $u_{1}>0$ is the first element of the vector $u\in \mathcal {R}^{n}$. Then we compute the Householder vector *v* as follows (Algorithm 1).

**Algorithm 1:**
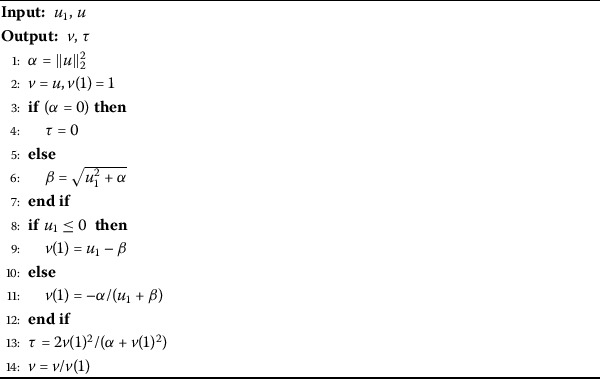
Computation of parameter *τ* and Householder vector *v* [12]

**Algorithm 2:**
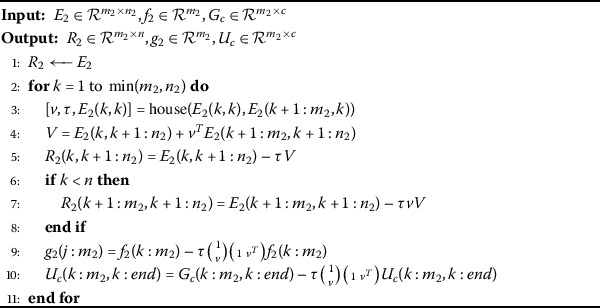
Calculating the *QR* factorization and computing matrix $U_{c}$

**Algorithm 3:**
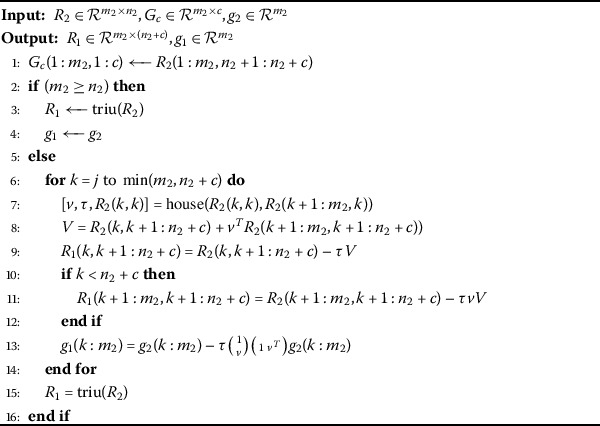
Updating $R_{2}$ factor after appending a block of columns $G_{c}$

**Algorithm 4:**
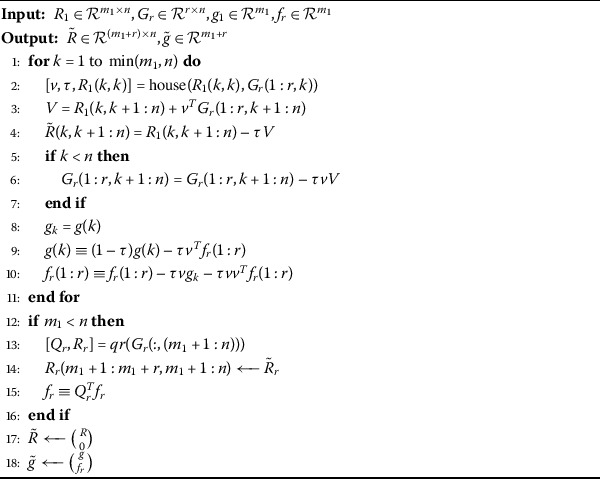
Updating $R_{1}$ factor after appending a block of rows $G_{r}$

**Algorithm 5:**
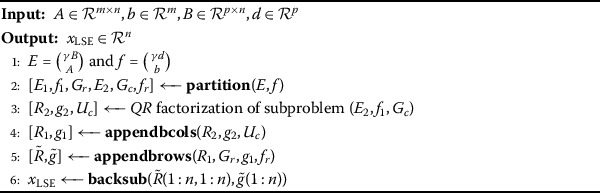
Updating *QR* algorithm for solution of LSE problem

## Updating *QR* factorization procedure for solution of LSE problem

Let

$$ E=\begin{pmatrix} \gamma B \\ A \end{pmatrix} \in R^{(m+p)\times n} \quad\text{and}\quad f=\begin{pmatrix} \gamma d \\ b \end{pmatrix} \in R^{m+p}. $$

Then we can write problem (3) as

7 $$ \min_{x(\gamma)} \Vert Ex-f \Vert _{2}. $$

Here, we will reduce problem (7) to a small incomplete subproblem using suitable partition process. In partition process, we remove blocks of rows and columns from the problem matrix *E* considered in (7) and from its corresponding right-hand side (RHS) without involving any arithmetics. That is, we consider

8 $$ E=\begin{pmatrix} E(1:j-1,1:n) \\ E(j:j+r,1:n) \\ E(j+r+1:m+p,1:n) \end{pmatrix} \quad\text{and}\quad f=\begin{pmatrix} f(1:j-1) \\ f(j:j+r) \\ f(j+r+1:m+p) \end{pmatrix}, $$

and removing blocks of rows from both *E* and *f* in equation (8) as

9 $$ G_{r}= E(j:j+r,1:n)\in \mathcal {R}^{r\times n} \quad\text{and}\quad f_{r}= f(j:j+r), $$

we get

10 $$ E_{1}=\begin{pmatrix} E(1:j-1,1:n) \\ E(j+r+1:m+p,1:n) \end{pmatrix}, \qquad f_{1}= \begin{pmatrix} f(1:j-1) \\ f(j+r+1:m+p) \end{pmatrix}. $$

Hence, we obtain the following problem:

11 $$ \min_{x_{1}} \Vert E_{1} x_{1}- f_{1} \Vert _{2}, \quad E_{1}\in \mathcal {R}^{{m_{1}}\times n_{1}}, f_{1}\in \mathcal {R}^{m_{1}}, x_{1}\in \mathcal {R}^{n_{1}}, $$

where $m_{1}=m+p-r$, $n_{1}=n$, and $f_{r}\in \mathcal {R}^{r}$. Furthermore, by removing block of columns $G_{c}=E_{1}(:,j: j+c)$ from the *j*th position by considering the partition of $E_{1}$ in the incomplete problem (11) as

12 $$ E_{2}= \begin{pmatrix} E_{1}(:,1:j-1) &E_{1}(:,j: j+c)& E_{1}(:,j+c+1:n) \end{pmatrix}, $$

we obtain the following reduced subproblem:

13 $$ \min_{x_{2}} \Vert E_{2}x_{2}- f_{2} \Vert _{2},\quad E_{2}\in \mathcal {R}^{m_{2}\times n_{2}}, f_{2}\in \mathcal {R}^{m_{2}}, x_{2}\in \mathcal {R}^{n_{2}}, $$

where $E_{2}= [E_{1}(:,1:j-1), E_{1}(:,j+1:n_{1}) ]$, $n_{2}=n_{1}-c$, $m_{2}=m_{1}$, and $f_{1}=f_{2}$.

Now, we calculated the *QR* factorization of the incomplete subproblem (13) is order to reduce it to the upper triangular matrix $R_{2}$ using the following algorithm (Algorithm 2).

Here house denotes the Householder algorithm and the Householder vectors are calculated using Algorithm 1 and *V* is a self-explanatory intermediary variable.

Hence, we obtain

14 $$ R_{2}=H_{n_{2}}\cdots H_{1} E_{2}, \qquad g_{2}= H_{n_{2}} \cdots H_{1} f_{2} $$

and

$$U_{c}= H_{n_{2}} \cdots H_{1} G_{c}. $$

Here, the *QR* factorization can also be obtained directly using the MATLAB built-in command *qr* but it is not preferable due to its excessive storage requirements for orthogonal matrix *Q* and by not necessarily providing positive sign of diagonal elements in the matrix $R_{2}$ [12].

To obtain the solution of problem (7), we need to update the upper triangular matrix $R_{2}$. For this purpose, we append the updated block of columns $G_{c}$ to the $R_{2}$ factor in (14) at the *j*th position as follows:

15 $$ R_{2c}= \begin{pmatrix} R_{2}(:,1:j-1) &G_{c}(:,j:j+c) & R_{2}(:,j+c+1:n) \end{pmatrix}. $$

Here, if the $R_{2}c$ factor in (15) is upper trapezoidal or in upper triangular form then no further calculation is required, and we get $R_{1}=R_{2c}$. Otherwise, we will need to reduce equation (15) to the upper triangular factor $R_{1}$ by introducing the Householder matrices $H_{n_{2}+c}, \ldots, H_{j}$:

16 $$ R_{1}=H_{n_{2}+c} \cdots H_{j} R_{2c}\quad \mbox{and}\quad g_{1}= H_{n_{2}+c} \cdots H_{j}g_{2}, $$

where $R_{1}\in \mathcal {R}^{m_{1}\times n_{1}}$ is upper trapezoidal for $m_{1}< n_{1}$ or it is an upper triangular matrix. The procedure for appending the block of columns and updating of the $R_{2}$ factor in algorithmic form is given as follows (Algorithm 3).

Here the term triu denotes the upper triangular part of the concerned matrix and *V* is the intermediary variable.

Now, we append a block of rows $G_{r}$ to the $R_{1}$ factor and $f_{r}$ to its corresponding RHS at the *j*th position in the following manner.

$$R_{r}=\begin{pmatrix} R_{1}(1:j-1,:) \\ G_{r}(1:r,:) \\ R_{1}(j:m_{1},:) \end{pmatrix},\qquad g_{r}= \begin{pmatrix} g_{1}(1:j-1) \\ f_{r}(1:r) \\ g_{1}(j:m_{1}) \end{pmatrix}. $$

We use the permutation matrix *P* in order to bring the block of rows $G_{r}$ and $f_{r}$ at the bottom position if required. Then

17 $$\begin{aligned} PR_{r}=\begin{pmatrix} R_{1} \\ G_{r} \end{pmatrix},\qquad Pg_{r}= \begin{pmatrix} g_{1} \\ f_{r} \end{pmatrix}. \end{aligned}$$

The equation (17) is reduced to upper triangular form by constructing the matrices $H_{1},\ldots,H_{n}$ using Algorithm 1 as given by

$$\tilde{R}=H_{n} H_{n-1}\cdots H_{1} \begin{pmatrix} R_{1} \\ G_{r} \end{pmatrix} $$

and

$$\tilde{g}=H_{n} H_{n-1} \cdots H_{1} \begin{pmatrix} g_{1} \\ f_{r} \end{pmatrix}. $$

The procedure is performed in algorithmic form as follows (Algorithm 4).

Here *qr* is the MATLAB command of *QR* factorization and *V* is a self-explanatory intermediary variable.

The solution of problem (7) can then be obtained by applying the MATLAB built-in command *backsub* for a back-substitution procedure.

The description of *QR* updating algorithm in compact form is given as follows (Algorithm 5).

Here, Algorithm 5 for a solution of LSE problem (1) calls upon the partition process, Algorithms 2, 3, 4 and MATLAB command *backsub* for back-substitution procedure, respectively.

## Error analysis

In this section, we will study the backward stability of our proposed Algorithm 5. The mainstay in our presented algorithm for the solution of LSE problem is the updating procedure. Therefore, our main concern is to study the error analysis of the updating steps. For others, such as the effect of using the weighting factor, finding the *QR* factorization and for the back-substitution procedure, we refer the reader to [28, 31]. Here, we recall some important results without giving their proofs and refer the reader to [28].

We will consider the following standard model of floating point arithmetic in order to analyze the rounding errors effects in the computations:

18 $$ fl(x \mbox{ op } y)=(x \mbox{ op } y) (1+\eta),\qquad \vert \eta \vert \le \epsilon_{M},\qquad \mbox{op}=+,-, *, /, $$

where $\epsilon_{M}$ is the unit roundoff. The value of $\epsilon_{M}$ is of order 10^−8^ in single precision computer arithmetic, while in double precision it is of the order 10^−16^. Moreover, for addition and subtraction in the absence of guard integers, we can write model (18) as follows:

$$ fl(x \pm y)=x (1+\eta_{1})\pm y (1+\eta_{2}),\qquad \vert \eta_{i} \vert \le\epsilon _{M},\quad i=1,2. $$

### Lemma 4.1

([28])

*If* $\vert \eta_{i} \vert \le\epsilon_{M}, \delta_{i}=\pm1\textit{ for }i=1,2,\ldots,n\textit{ and }n \epsilon_{M} < 1$, *then*

$$ \prod_{i=1}^{n}(1+\eta_{i})^{\delta_{i}} =1+\phi_{n}, $$

*where*

$$ \vert \phi_{n} \vert \le\frac{n\epsilon_{M}}{1-n\epsilon_{M}}=\gamma_{n}. $$

Here, we will use the constants $\gamma_{n}$ and $\tilde{\gamma}_{cn}$ for convenience as adopted in [28] where these are defined by

19 $$ \gamma_{n}=\frac{n\epsilon_{M}}{1-n\epsilon_{M}}, \quad\mbox{assuming } n \epsilon_{M}< 1, $$

and

20 $$ \tilde{\gamma}_{cn}=\frac{nc\epsilon_{M}}{1-nc\epsilon_{M}}, \quad\mbox{assuming } nc\epsilon_{M}< 1, $$

where *c* is a small positive integer.

### Lemma 4.2

([28])

*Let* $x, y\in \mathcal {R}^{n}$ *and consider the inner product* $x^{T} y$. *Then*

$$ fl\bigl(x^{T} y\bigr)=x^{T}(y+\triangle y),\qquad \vert \triangle y \vert \leq\gamma_{n} \vert y \vert . $$

### Lemma 4.3

([28])

*Let* $\phi_{k}$ *and* $\gamma_{k}$ *be defined for any positive integer* *k*. *Then for positive integers* *j* *and* *k* *the following relations hold*:

21 $$\begin{aligned} &(1+\phi_{k}) (1+\phi_{j})=1+ \phi_{k+j}, \end{aligned}$$

22 $$\begin{aligned} &\frac{(1+\phi_{k})}{(1+\phi_{j})}= \textstyle\begin{cases}1+\phi_{k+j}, & j \leq k,\\ 1+\phi_{k+2j},& j>k, \end{cases}\displaystyle \end{aligned}$$

23 $$\begin{aligned} &j\gamma_{k} \leq\gamma_{jk}, \end{aligned}$$

24 $$\begin{aligned} &k\gamma_{k}+\epsilon_{M} \leq \gamma_{k+1}, \end{aligned}$$

25 $$\begin{aligned} &\gamma_{k}+\gamma_{j}+\gamma_{k} \gamma_{j}\leq \gamma_{k+j}. \end{aligned}$$

### Lemma 4.4

([28])

*Considering the construction of* $\tau\in \mathcal {R}$ *and* $v\in \mathcal {R}^{n}$ *given in Section* 2, *then the computed* *τ̃* *and* *ṽ* *in floating point arithmetic satisfy*

$$ \tilde{v}(2:n)=v(2:n) $$

*and*

$$ \tilde{\tau}=\tau(1+\tilde{\phi}_{n})\quad\textit{and}\quad\tilde {v_{1}}=v_{1}(1+\tilde{\phi}_{n}), $$

*where* $\tilde{\phi}_{n}\leq\tilde{\gamma}_{n}$.

Here, we represent the Householder transformation as $I-vv^{T}$, which requires $\Vert v \Vert _{2}=\sqrt{2}$. Therefore, by redefining $v=\sqrt {\tau}v$ and $\tau=1$ using Lemma 4.4, we have

26 $$ \tilde{v}=v+\triangle v,\qquad \vert \triangle v \vert \leq\tilde{ \gamma}_{m}v \quad\text{for } v\in \mathcal {R}^{m}, \Vert v \Vert _{2}=\sqrt{2}. $$

### Lemma 4.5

([29])

*Considering the computation of* $y=\tilde{H}b=(I-\tilde{v} \tilde {v}^{T})b=b-\tilde{v}(\tilde{v}^{T}b)$ *for* $b\in \mathcal {R}^{m}$ *and* $\tilde {v}\in \mathcal {R}^{m}$ *satisfies* (26). *Then the computed* *ỹ* *satisfies*

$$ \tilde{y}=(H+\triangle H)b, \qquad \Vert \triangle H \Vert _{F}\leq \tilde{\gamma}_{m}, $$

*where* $H=I-vv^{T}$ *and* $\Vert \cdot \Vert _{F}$ *denotes the Frobenius norm*.

### Lemma 4.6

([28])

*Let* $H_{k}=I-v_{k} v_{k}^{T}\in \mathcal {R}^{m\times m}$ *be the Householder matrix and we define the sequence of transformations*

$$ X_{k+1}=H_{k} X_{k}, \quad k=1:r, $$

*where* $X_{1}= X\in\mathcal{R}^{m\times n}$. *Furthermore*, *it is assumed that these transformations are performed using computed Householder vectors* $\tilde{v}_{k}\approx v_{k}$ *and satisfy Lemma* 4.4. *Then we have*

27 $$ \tilde{X}_{r+1}=Q^{T}(X+ \triangle X), $$

*where* $Q^{T}=H_{r},\ldots,H_{1}$, *and*

$$\Vert \triangle x_{j} \Vert _{2} \leq r\tilde{\gamma }_{m} \Vert x_{j} \Vert _{2},\quad j=1:n. $$

### Theorem 4.7

([28])

*Let* $\tilde{R}\in\mathcal{R}^{m\times n}$ *be the computed upper trapezoidal* *QR* *factor of* $X\in\mathcal{R}^{m\times n}\ (m\geq n)$ *obtained via Householder* *QR* *algorithm by Lemma* 4.6. *Then there exists an orthogonal matrix* $Q\in \mathcal{R}^{m\times m}$ *such that*

$$ X+\triangle X=Q\tilde{R}, $$

*where*

28 $$ \Vert \triangle x_{j} \Vert _{2}\leq \tilde{\gamma }_{mn} \Vert x_{j} \Vert _{2},\quad j=1:n. $$

### Lemma 4.8

([28])

*Let* $X\in\mathcal{R}^{m\times n}$ *and* $H=I-\tau v v^{T}\in R^{m\times m}$ *be the Householder matrix*. *Also*, *assuming that the computation of* *HX* *is performed using computed* *τ̃* *and* *ṽ* *such that it satisfies Lemma* 4.4. *Then*, *from Theorem* 4.7, *we have*

29 $$ fl(HX)=H(X+\triangle X),\qquad \Vert \triangle X \Vert _{F} \leq \gamma_{cm} \Vert X \Vert _{F}. $$

### Backward error analysis of proposed algorithm

To appreciate the backward stability of our proposed Algorithm 5, we first need to carry out the error analysis of Algorithms 3 and 4. For this purpose, we present the following.

#### Theorem 4.9

*The computed factor* *R̃* *in Algorithm* 4 *satisfies*

$$ \tilde{R}=Q^{T} \left[\begin{pmatrix} R_{1} \\ G_{r} \end{pmatrix}+ e_{r} \right] \quad\textit{and}\quad \Vert e_{r} \Vert _{F}\leq\tilde{\gamma }_{n(r+1)} \left \Vert \begin{pmatrix} R_{1} \\ G_{r} \end{pmatrix} \right \Vert _{F}, $$

*where* $G_{r}\in \mathcal {R}^{r\times n}$ *is the appended block of rows to the* $R_{1}$ *factor and* $Q=H_{1} H_{2}\cdots H_{n}$.

#### Proof

Let the Householder matrix $H_{j}$ have zeros on the *j*th column of the matrix $\bigl({\scriptsize\begin{matrix}{} \tilde{r}_{jj} \cr G_{rj} \end{matrix}}\bigr)$. Then using Lemma 4.6, we have

30 $$ H_{1} \left[\begin{pmatrix} R_{1} \\ G_{r} \end{pmatrix}+e_{1} \right]=\begin{pmatrix} \tilde{R}_{1r} \\ \tilde{G}_{1r} \end{pmatrix}, $$

where

$$ \Vert e_{1} \Vert _{F}\leq\tilde{\gamma}_{r+1} \left \Vert \begin{pmatrix} R_{1} \\ G_{r} \end{pmatrix} \right \Vert _{F}. $$

Similarly,

$$\begin{aligned} H_{2} \left[\begin{pmatrix} \tilde{R}_{1r}\\ \tilde{G}_{1r} \end{pmatrix}+e_{2} \right]&= \begin{pmatrix} \tilde{R}_{2r} \\ \tilde{G}_{2r} \end{pmatrix} \\ &=H_{2} H_{1} \left[\begin{pmatrix} R_{1} \\ G_{r} \end{pmatrix}+e_{r}^{(2)} \right], \end{aligned}$$

and

$$ \Vert e_{2} \Vert _{F}\leq\tilde{\gamma}_{r+1} \left \Vert \begin{pmatrix} \tilde{R}_{1r}\\ \tilde{G}_{1r} \end{pmatrix} \right \Vert _{F}, $$

where

$$\begin{aligned} \bigl\Vert {e_{r}^{(2)}} \bigr\Vert _{F}&= \Vert e_{1}+e_{2} \Vert _{F}, \\ \bigl\Vert e_{r}^{(2)} \bigr\Vert _{F} &\leq \Vert e_{1} \Vert _{F}+ \Vert e_{2} \Vert _{F}, \\ &\leq\tilde{\gamma}_{r+1} \left \Vert \begin{pmatrix} R_{1} \\ G_{r} \end{pmatrix} \right \Vert _{F}+\tilde{\gamma}_{r+1} \left \Vert \begin{pmatrix}\hat{R}_{1r}\\ \hat{G}_{1r} \end{pmatrix} \right \Vert _{F}. \end{aligned}$$

From equation (30), we have

$$\begin{aligned} \left \Vert \begin{pmatrix} \hat{R}_{1r}\\ \hat{G}_{1r} \end{pmatrix} \right \Vert _{F}&\leq \left \Vert \begin{pmatrix} R_{1} \\ G_{r} \end{pmatrix} \right \Vert _{F}+e_{1} \\ &\leq \left \Vert \begin{pmatrix} R_{1} \\ G_{r} \end{pmatrix} \right \Vert _{F}+\tilde{ \gamma}_{r+1} \left \Vert \begin{pmatrix} R_{1} \\ G_{r} \end{pmatrix} \right \Vert _{F}. \end{aligned}$$

This implies that

$$\begin{aligned} \bigl\Vert {e_{r}^{(2)}} \bigr\Vert _{F} & \leq\tilde{\gamma}_{r+1} \left \Vert \begin{pmatrix} R_{1} \\ G_{r} \end{pmatrix} \right \Vert _{F}+\tilde{\gamma}_{r+1}(1+\tilde{\gamma }_{r+1}) \left \Vert \begin{pmatrix} R_{1} \\ G_{r} \end{pmatrix} \right \Vert _{F} \\ & \leq\bigl(\tilde{\gamma}_{r+1}+\tilde {\gamma }_{r+1}(1+ \tilde{\gamma}_{r+1})\bigr) \left \Vert \begin{pmatrix} R_{1} \\ G_{r} \end{pmatrix} \right \Vert _{F} \\ & \leq2\tilde{\gamma}_{r+1} \left \Vert \begin{pmatrix} R_{1} \\ G_{r} \end{pmatrix} \right \Vert _{F}, \end{aligned}$$

where we ignored $(\tilde{\gamma}_{r+1})^{2}$ as $\tilde{\gamma }_{r+1}$ is very small.

Continuing in the same fashion till the *n*th Householder reflection, we have

$$ \tilde{R}=H_{n} H_{n-1}\cdots H_{1} \left[ \begin{pmatrix} R_{1} \\ G_{r} \end{pmatrix}+e_{r} \right], $$

where

$$ \Vert e_{r} \Vert _{F}= \bigl\Vert e_{r}^{(n)} \bigr\Vert _{F}\leq n\tilde{ \gamma}_{r+1} \left \Vert \begin{pmatrix} R_{1} \\ G_{r} \end{pmatrix} \right \Vert _{F}, $$

or, by using equation (23) of Lemma 4.3, we can write

$$ \Vert e_{r} \Vert _{F}\leq\tilde{\gamma}_{n(r+1)} \left \Vert \begin{pmatrix} R_{1} \\ G_{r} \end{pmatrix} \right \Vert _{F}, $$

which is the required result. □

#### Theorem 4.10

*The backward error for the computed factor* $R_{1}$ *in Algorithm* 3 *is given by*

$$ R_{1}=\tilde{Q}^{T} \bigl[(R_{2},U_{c})+ \tilde{e_{c}} \bigr], $$

*where* $U_{c}\in \mathcal {R}^{(m+p)\times c}$ *is the appended block of columns to the right*-*end of* $R_{2}$ *factor*, $\Vert \tilde{e}_{c} \Vert _{F}\leq\tilde {\gamma}_{(m+p)c} \Vert U_{c} \Vert _{F}$ *and* $\tilde {Q}=H_{n_{2}+1}\cdots H_{n_{2}+c}$.

#### Proof

To prove the required result, we proceed similar to the proof of Theorem 4.9, and obtain

$$ \bigl\Vert e_{c}^{2} \bigr\Vert _{F} \leq2 \tilde{\gamma}_{c1} \bigl\Vert \bigl[(R_{2},U_{c})+ \tilde{e_{c}} \bigr] \bigr\Vert _{F}. $$

This implies that the error in the whole process of appending the block of columns to the $R_{2}$ factor is given by

$$\begin{aligned} R_{1}=H_{n_{2}+c} \cdots H_{n_{2}+1} \bigl[(R_{2},U_{c})+\tilde {e_{c}} \bigr], \end{aligned}$$

where

$$ \Vert e_{c} \Vert _{F}\leq\tilde{\gamma}_{(m+p)c} \Vert U_{c} \Vert _{F}. $$

 □

#### Theorem 4.11

*Let the LSE problem* (1) *satisfy the conditions given in* (2). *Then Algorithm *5 *solves the LSE problem with computed* $\tilde{Q}=H_{1}\cdots H_{n}$ *and* *R̃* *which satisfies*

31 $$ \bigl\Vert I-\tilde{Q}^{T} \tilde{Q} \bigr\Vert _{F} \leq\sqrt{n} \tilde{\gamma}_{(m+p)n} $$

*and*

32 $$ \Vert E-\tilde{Q} \tilde{R} \Vert _{F}\leq\sqrt{n} \tilde{\gamma }_{(m+p)n} \Vert E \Vert _{F}. $$

#### Proof

To study the backward error analysis of Algorithm 5, we consider the reduced subproblem matrix $E_{2}\in \mathcal {R}^{m_{2}\times n_{2}}$ from (13) and let $\hat{e}_{2}$ be its backward error in the computed *QR* factorization. Then from Lemma 4.8, we have

$$ Q_{2}^{T} (E_{2}+\hat{e}_{2})= R_{2} \quad\mbox{and}\quad \Vert \hat {e}_{2} \Vert _{F}\leq\tilde{\gamma}_{m_{2}n_{2}} \Vert E_{2} \Vert _{F}. $$

As in our proposed Algorithm 5, we appended a block of columns $U_{c}=Q_{2}^{T} G_{c}$ to the $R_{2}$ factor, then by using Theorem 4.10, we have

$$ R_{1}=H_{n_{2}+1}\cdots H_{n_{2}+c} \bigl[(R_{2},U_{c})+e_{c} \bigr], $$

where $\Vert e_{c} \Vert _{F}\leq\tilde{\gamma }_{m_{2}c} \Vert U_{c} \Vert _{F}$.

Therefore, by simplifying

$$ \Vert \hat{e}_{1} \Vert _{F}= \bigl\Vert Q_{1}^{T} Q_{2}^{T} \hat {e}_{2}+ Q_{1}^{T} e_{c} \bigr\Vert _{F}, $$

where $Q_{1}^{T}=H_{n_{2}+1}\cdots H_{n_{2}+c}$ and $\hat{e}_{2}$ is the error of computing the *QR* factorization, we obtain

$$\begin{aligned} \Vert \hat{e}_{1} \Vert _{F}&= \bigl\Vert Q_{1}^{T} Q_{2}^{T} \hat {e}_{2}+ Q_{1}^{T} e_{c} \bigr\Vert _{F} \\ &\leq \bigl\Vert Q_{1}^{T} \bigr\Vert _{F} \bigl\Vert Q_{2}^{T} \bigr\Vert _{F} \Vert \hat{e}_{2} \Vert _{F}+ \bigl\Vert Q_{1}^{T} \bigr\Vert _{F} \Vert e_{c} \Vert _{F} \\ &\leq \Vert \hat{e}_{2} \Vert _{F}+ \Vert e_{c} \Vert _{F} \\ &\leq\tilde{\gamma}_{m_{2}n_{2}} \Vert E_{2} \Vert _{F}+\tilde {\gamma }_{m_{2}c} \Vert U_{c} \Vert _{F} \\ &\leq[\tilde{\gamma}_{m_{2}n_{2}}+\tilde{\gamma}_{m_{2}c}]\mbox{max} \bigl({ \Vert E_{2} \Vert _{F}, \Vert U_{c} \Vert _{F}} \bigr). \end{aligned}$$

Hence, we have

$$ \Vert \hat{e}_{1} \Vert _{F}\leq[\tilde{\gamma }_{m_{2}n_{2}}+\tilde{\gamma }_{m_{2}c}]\mbox{max} \bigl({ \Vert E_{2} \Vert _{F}, \Vert U_{c} \Vert _{F}} \bigr), $$

which is the total error at this stage of appending the block of columns and its updating. Furthermore, we appended the block of rows $G_{r}$ to the computed factor $R_{1}$ in our algorithm and, by using Theorem 4.9, we obtain

$$ \tilde{R}=H_{n}\cdots H_{1} \left[\begin{pmatrix} R_{1} \\ G_{r} \end{pmatrix}+e_{r} \right], $$

where

$$ \Vert e_{r} \Vert _{F} \leq\tilde{ \gamma}_{(r+1)n} \left \Vert \begin{pmatrix} R_{1} \\ G_{r} \end{pmatrix} \right \Vert _{F}, $$

is the error for appending the block of rows $G_{r}$.

Hence, the total error *e* for the whole updating procedure in Algorithm 5 can be written as

33 $$\begin{aligned} \Vert e \Vert _{F}& \leq \Vert e_{r} \Vert _{F}+ \Vert \hat{e}_{1} \Vert _{F} \\ &\leq\tilde{\gamma}_{(r+1)n} \left \Vert \begin{pmatrix}R_{1} \\ G_{r} \end{pmatrix} \right \Vert _{F}+(\tilde{\gamma}_{m_{2}n_{2}}+ \tilde{\gamma }_{m_{2}c})\mbox{max} \bigl({ \Vert E_{2} \Vert _{F}, \Vert U_{c} \Vert _{F}} \bigr) \\ &\leq(\tilde{\gamma}_{(r+1)n}+\tilde{\gamma}_{m_{2}n_{2}}+ \tilde { \gamma}_{m_{2}c})\mbox{max} \left({ \Vert E_{2} \Vert _{F}, \Vert U_{c} \Vert _{F}}, \left \Vert \begin{pmatrix}R_{1} \\ G_{r} \end{pmatrix} \right \Vert _{F} \right). \end{aligned}$$

Now, if the orthogonal factor *Q* is to be formed explicitly, then the deviation from normality in our updating procedure can be examined. For this, we consider $E=I$, then from Lemma 4.8, we have

$$ \tilde{Q}_{2}=Q_{2}^{T}(I_{m_{2}}+\zeta), $$

where *ζ* is the error in the computed factor $\tilde{Q}_{2}$ given as

$$ \Vert \zeta \Vert _{F}\leq\tilde{\gamma}_{m_{2} n_{2}} \Vert I_{m_{2}} \Vert _{F}=\sqrt {n_{2}} \tilde{ \gamma}_{m_{2} n_{2}}, $$

where $\Vert I_{m_{2}} \Vert _{F}=\sqrt{n_{2}}$. In a similar manner, the computed factor $\tilde{Q}_{1}$ after appending columns $U_{c}$ is given by

$$ \tilde{Q}_{1}=Q_{1}^{T}(I_{m_{2}}+ \zeta_{c}), $$

where

$$ \Vert \zeta_{c} \Vert _{F}\leq\sqrt{c}\tilde{ \gamma}_{m_{2}c}. $$

Therefore, the total error in *Q̃* is given as

$$ \tilde{Q_{2}}\tilde{Q_{1}}=Q_{2}^{T}(I_{m_{2}}+ \zeta_{1}), $$

where

$$ \Vert \zeta_{1} \Vert _{F}\leq \Vert \zeta _{2} \Vert _{F}+ \Vert \zeta_{c} \Vert _{F} \leq \sqrt{n_{2}} \tilde{\gamma}_{m_{2} n_{2}}+ \sqrt{c}\tilde{\gamma}_{m_{2} c}. $$

Similarly, the error in the computed factor *Q̃* after appending block of rows is given as

$$ \tilde{Q}=Q^{T}(I_{r}+\zeta_{r}), $$

where

$$ \Vert \zeta_{r} \Vert _{F}\leq\sqrt{n} \tilde{ \gamma}_{(r+1)n}. $$

So, the total error *ζ* in *Q* during the whole updating procedure is given by

34 $$\begin{aligned} \Vert \zeta \Vert _{F}&= \Vert \zeta _{1} \Vert _{F}+ \Vert \zeta_{r} \Vert _{F} \\ &\leq\sqrt{n} \tilde{\gamma}_{(r+1)n}+\sqrt{n_{2}}\tilde{\gamma }_{m_{2}n_{2}}+\sqrt{c} \tilde{\gamma}_{m_{2}c}, \end{aligned}$$

where $m_{2}< m+p$, $n_{2}< n$ and $c< n$.

Therefore, the error measure in *Q̃* which shows the amount by which it has been deviated from normality is given by

35 $$ \bigl\Vert I-\tilde{Q}^{T} \tilde{Q} \bigr\Vert _{F} \leq\sqrt {n}(\tilde{\gamma }_{(r+1)n}+\tilde{ \gamma}_{m_{2}n_{2}}+ \tilde{\gamma}_{m_{2}c}). $$

From equation (20), we have

36 $$ \tilde{\gamma}_{(r+1)n}+\tilde{\gamma}_{m_{2}n_{2}}+ \tilde{\gamma }_{m_{2}c}\approx\tilde{\gamma}_{(m+p+1)n}=\tilde{ \gamma}_{(m+p)n}, $$

and using it in equation (35), we get the required result (31).

Also, we have

37 $$\begin{aligned} \Vert E-\tilde{Q} \tilde{R} \Vert _{F}&= \bigl\Vert (E-Q\tilde{R}) +\bigl((Q-\tilde{Q})\tilde{R}\bigr) \bigr\Vert _{F} \\ &\leq\sqrt{n}(\tilde{\gamma}_{(r+1)n}+\tilde{\gamma }_{m_{2}n_{2}}+ \tilde{\gamma}_{m_{2}c})\max \left({ \Vert E_{2} \Vert _{F}, \Vert E_{c} \Vert _{F}}, \left \Vert \begin{pmatrix}R_{1} \\ G_{r} \end{pmatrix} \right \Vert _{F} \right). \end{aligned}$$

As $\Vert X \Vert _{F}= \Vert \mathit{QR} \Vert _{F}= \Vert R \Vert _{F}$, therefore, we can write

38 $$ \max \left({ \Vert E_{2} \Vert _{F}, \Vert U_{c} \Vert _{F}}, \left \Vert \begin{pmatrix}R_{1} \\ G_{r} \end{pmatrix} \right \Vert _{F} \right)= \left \Vert \begin{pmatrix} R_{1} \\ G_{r} \end{pmatrix} \right \Vert _{F}= \Vert E \Vert _{F}. $$

Hence, applying expressions (36) and (38) to (37), we obtain the required equation (32) which shows how large is the error in the computed *QR* factorization. □

## Numerical experiments

This section is devoted to some numerical experiments which illustrate the applicability and accuracy of Algorithm 5. The problem matrices *A* and *B* and its corresponding right-hand side vectors *b* and *d* are generated randomly using the MATLAB built-in commands $\operatorname{rand} (\mbox{`}\mathrm{twister}\mbox{'})$ and rand. These commands generate pseudorandom numbers from a standard uniform distribution in the open interval $(0,1)$. The full description of test matrices are given in Table 1, where we denote the Frobenius norm with $\Vert \cdot \Vert _{F}$ and the condition number by $\kappa(\cdot)$. For accuracy of the solution, we consider the actual solution such that $x=\operatorname{rand}(n,1)$ and denote the result obtained from Algorithm 5 by $x_{\mathrm{LSE}}$ and that of direct *QR* Householder factorization with column pivoting by $x_{p}$. We obtain the relative errors between the solutions given in Table 2. Moreover, the solution $x_{\mathrm{LSE}}$ satisfy the constrained system effectively. The description of the matrix *E*, the size of the reduced subproblem (SP), value of the weighted factor *ω*, the relative errors $\operatorname{err}= \Vert x-x_{\mathrm{LSE}} \Vert _{2}/ \Vert x \Vert _{2}$ and $\operatorname {err1}= \Vert x-x_{p} \Vert _{2}/ \Vert x \Vert _{2}$ are provided in Table 2. We also carry out the backward error tests of Algorithm 5 numerically for our considered problems and provide the results in Table 3, which agrees with our theoretical results.

**Table 1:** **Description of test problems**

**Problem**	**Size of (A)**	***κ*** **(** ***A*** **)**	$\boldsymbol {\Vert A \Vert _{F}}$	**Size of (B)**	***κ*** **(** ***B*** **)**	$\boldsymbol { \Vert B \Vert _{F}}$
1.	10×8	1.3667e+02	2.0006e+02	6×8	7.4200e+01	1.0216e+02
2.	100×90	2.9303e+03	2.1395e+03	90×90	3.3687e+03	1.3735e+03
3.	800×700	6.2106e+03	1.6872e+04	600×700	1.6164e+03	9.9000e+03
4.	1,000×500	1.1602e+03	1.5943e+04	500×500	1.2883e+05	7.6360e+03
5.	2,000×1,000	1.6727e+03	3.1884e+04	1,000×1,000	1.7430e+06	1.5272e+04

**Table 2:** **Results comparison**

**Problem**	**Size of (E)**	***γ***	**Size of SP**	**err1**	**err**
1.	16×8	8.9564e+15	3×3	1.3222e−14	1.4585e−15
2.	190×90	7.1258e+15	3×3	1.2628e−13	5.5294e−14
3.	1,400×700	7.7993e+15	3×3	1.2821e−12	4.2522e−13
4.	1,500×500	9.5551e+15	3×3	1.9377e−12	1.3559e−12
5.	3,000×1,000	9.5549e+15	3×3	1.0828e−10	8.5181e−12

**Table 3:** **Backward error analysis results**

**Problem**	$\boldsymbol {\frac{ \Vert E-\tilde{Q} \tilde{R} \Vert _{F}}{ \Vert E \Vert _{F}}}$	$\boldsymbol {\Vert I-\tilde{Q}^{T} \tilde{Q} \Vert _{F}}$
1.	4.4202e−16	1.3174e−15
2.	4.7858e−16	9.0854e−15
3.	1.0450e−15	4.9428e−14
4.	9.0230e−16	3.8711e−14
5.	9.9304e−16	6.4026e−14

## Conclusion

The solution of linear least squares problems with equality constraints is studied by updated techniques based on *QR* factorization. We updated only the *R* factor of the *QR* factorization of the small subproblem in order to obtain the solution of our considered problem. Numerical experiments are provided which illustrated the accuracy of the presented algorithm. We also showed that the algorithm is backward stable. The presented approach is suitable for dense problems and also applicable where *QR* factorization of a problem matrix is available and we are interested in the solution after adding new data to the original problem. In the future, it will be of interest to study the updating techniques for sparse data problems and for those where the linear least squares problem is fixed and the constraint system is changing frequently.
